# Oncological Horizons: The Synergy of Medical and Surgical Innovations in Cancer Treatment

**DOI:** 10.7759/cureus.49249

**Published:** 2023-11-22

**Authors:** Muhammad Shams, Shenouda Abdallah, Lara Alsadoun, Yusra H Hamid, Rayan Gasim, Ahmad Hassan

**Affiliations:** 1 Urology, Royal Bournemouth Hospital, Bournemouth, GBR; 2 Surgery, Jaber Al-Ahmad Hospital, Kuwait, KWT; 3 Trauma and Orthopaedics, Chelsea and Westminster Hospital, London, GBR; 4 Community Medicine, Faculty of Medicine, University of Khartoum, Khartoum, SDN; 5 Internal Medicine, University of Khartoum, Khartoum, SDN; 6 Internal Medicine, Mayo Hospital, Lahore, PAK

**Keywords:** immunotherapy, targeted therapies, chemotherapy, historical progression, cancer treatment, surgical innovations, medical innovations, synergy, horizons, oncological

## Abstract

The landscape of cancer treatment has witnessed a remarkable transformation in recent years, marked by the convergence of medical and surgical innovations. Historically, cancer therapy faced challenges, including limited efficacy and severe side effects. This narrative review explores the historical progression of cancer treatments, shedding light on significant breakthroughs in both medical and surgical oncology. It comprehensively addresses the medical domain, covering chemotherapy, targeted therapies, immunotherapy, hormonal treatments, and radiological procedures. Simultaneously, it delves into the surgical realm, discussing the evolution of surgical techniques, minimally invasive procedures, and the role of surgery across various stages of cancer. The article emphasizes the fusion of medical and surgical approaches, highlighting neoadjuvant and adjuvant therapies and the significance of multidisciplinary tumor boards. It also addresses innovations, challenges, and the pivotal role of patient-centered care. Furthermore, it offers insights into the future directions and forecasts in the constantly evolving field of integrated oncological care. This review provides a comprehensive understanding of the dynamic and transformative nature of cancer treatment, reflecting the unwavering commitment of the medical and surgical communities in the ongoing fight against cancer.

## Introduction and background

The landscape of cancer treatment has undergone a remarkable transformation characterized by the convergence of medical and surgical innovations. Historically, cancer therapy has been a tumultuous voyage marked by challenges, including limited treatment efficacy and severe side effects [1]. Yet, it has also offered glimpses of hope and instances of complete remission. Traditionally, surgery has played a central role in managing solid tumors. However, recent years have witnessed a paradigm shift towards a more personalized approach known as precision oncology. This approach tailors treatments to individual patients based on their genomic alterations and cancer-specific molecular pathways, ensuring the precise administration of the right drug to the right patient at the right time [2].

Immunotherapy, an emerging field in cancer treatment, has gained prominence due to its specificity, adaptability, and durability in enhancing the immune system's response against cancer [3]. Nanotechnology has also entered the arena, offering nanostructures for controlled drug delivery, combining imaging and treatment, hyperthermia application, and targeted therapy. These therapies can be applied independently or in conjunction with other elements like antibodies and peptides. Additionally, gene therapy holds promise as an innovative treatment method [1].

Cancer care demands the specialized expertise of physicians such as medical oncologists, surgeons, and radiation oncologists [4]. Surgical resection remains a fundamental method for achieving control over primary tumors at the local and regional levels. The combination of surgery with adjuvant systemic treatments and radiotherapy holds substantial curative potential [5].

This review provides an in-depth exploration of the historical progression of cancer treatments, shedding light on significant breakthroughs in both medical and surgical oncology. It comprehensively addresses the medical domain, delving into topics such as chemotherapy, targeted therapies, immunotherapy, hormonal treatments, and radiological procedures. Concurrently, it delves into the surgical realm, covering the evolution of surgical techniques, minimally invasive procedures, and the role of surgery across various stages of cancer. The article underscores the fusion of medical and surgical approaches, accentuating neoadjuvant and adjuvant therapies and the significance of multidisciplinary tumor boards. It also addresses innovations, obstacles, and the pivotal role of patient-centric care. Finally, it offers insights into future directions and forecasts in the constantly evolving field of integrated oncological care.

## Review

Historical overview

Ancient Egyptian and Greek civilizations treated cancer with radical surgeries but achieved limited success. The late 1800s marked a turning point with the discovery of X-rays, beginning modern medical oncology. After World War II, cytotoxic anti-tumor drugs transformed the field, spurring significant research growth. In the 1980s, molecular-targeted therapies emerged, improving patient survival. Genetic engineering in the new millennium propelled oncology further. Monoclonal antibodies and immune checkpoint inhibitors offered hope to patients. Today, cancer research focuses on innovative cell therapies, anti-tumor vaccines, and biotechnological drugs, promising to redefine cancer treatment [6].

Surgery's historical involvement in oncology, spanning thousands of years, was characterized by limited success and elevated rates of morbidity and mortality until around 150 years ago when anesthesia and antiseptic methods were introduced. In the past five decades, a notable shift in approach has taken place, highlighting the importance of multimodal therapy, technological advancements, and minimally invasive techniques to reduce the necessity for radical surgical procedures. Today, a cohort of highly skilled medical professionals, including surgeons, oncologists, radiologists, scientists, anesthetists, and nurses, has metamorphosed cancer surgeries into routine, secure, and efficient practices, with a primary focus on preserving patients' physical integrity, functionality, and overall quality of life, all while maintaining their chances of survival [7].

For a long time, surgery has stood as the fundamental treatment modality for solid tumors, governed by fundamental oncological principles that have stood the test of time. Nevertheless, the last few decades have seen a revolution propelled by a deeper comprehension of the disease, advanced diagnostic capabilities, new or enhanced supplementary treatments, and cutting-edge surgical techniques that challenge established norms. This evolving landscape represents a meticulous reevaluation of historical methods and the amalgamation of discoveries from both fundamental and clinical research into an ever-expanding reservoir of knowledge [8].

The medical approach to oncology

Oncology has witnessed significant progress in recent years, reflecting a multifaceted strategy to combat this intricate and diverse disease. Chemotherapy, a foundational pillar of cancer treatment, employs toxic compounds to target rapidly dividing cancer cells. Its development can be traced back to the discovery of mustard gas's destructive impact on lymphatic tissues and bone marrow, paving the way for the use of nitrogen mustard derivatives. Today, chemotherapy encompasses a wide range of drugs, including polyadenosine diphosphate-ribose polymerase inhibitors, angiogenesis inhibitors, and histone deacetylase inhibitors, targeting various aspects of cancer cell biology [9].

In the era of precision medicine, targeted therapies have gained prominence, revolutionizing the landscape of medical oncology. These therapies capitalize on patient-specific clinical and molecular characteristics, offering tailored treatments that minimize exposure to unnecessary or ineffective therapies. The accessibility of patient and tumor genomics has enabled the identification and targeting of major molecular drivers of cancer, placing precision oncology at the forefront of treatment strategies. These advancements have the potential to enhance the effectiveness of cancer treatment while reducing side effects [10].

Immunotherapy, another innovative approach, leverages the body's immune system to recognize and attack cancer cells. Unlike traditional treatments that mainly target primary tumors, immunotherapy focuses on eradicating remaining malignant cells and tumor metastases. Techniques such as immune checkpoint inhibitors, chimeric antigen receptor (CAR) T-cell therapy, and cancer vaccines have emerged as powerful tools in the fight against cancer, offering a more natural and holistic approach to controlling disease progression [11].

Delving into the historical timelines of these immunotherapies offers valuable insight into their evolution in cancer treatment. Immune checkpoint inhibitors, which revolutionized the approach to activating the immune system against cancer, marked a significant milestone with the FDA approval of ipilimumab, an anti-CTLA-4 antibody, in 2011 for treating melanoma. This was followed by the development of programmed death-1 (PD-1)/programmed death-ligand 1 (PD-L1) inhibitors, further expanding the arsenal against various cancers. CAR T-cell therapy, involving the genetic modification of a patient's T-cells to target cancer cells, saw its foundational research in the late 1980s and early 1990s, leading up to the first FDA approval of such therapy (tisagenlecleucel) in 2017 for certain pediatric and young adult patients with B-cell acute lymphoblastic leukemia. The concept of cancer vaccines, bifurcated into preventive (like human papillomavirus (HPV) vaccines) and therapeutic vaccines, achieved a critical breakthrough with the approval of sipuleucel-T (Provenge) in 2010 for prostate cancer, marking the arrival of the first therapeutic cancer vaccine. These timelines underscore the progressive integration of immunotherapies into oncological practice, highlighting their transformative impact on cancer treatment strategies.

Hormonal therapy plays a crucial role in hormone-dependent malignancies by manipulating the endocrine system through the administration of exogenous hormones. This approach interferes with hormone production or receptor activity, affecting the growth of cancer cells. Imaging is integral in monitoring treatment response and detecting potential side effects. Hormonal therapy, like chemotherapy, can lead to decreases in tumor size, but it also comes with a spectrum of complications, making close monitoring and early intervention essential [12].

Interventional radiology is a vital component of oncology, offering treatments like transarterial embolization and percutaneous thermal ablations. Transarterial embolization, such as transarterial chemoembolization (TACE) or selective internal radiation therapy (SIRT), is often used for primary and secondary hepatic malignancies. Percutaneous thermal ablation is a minimally invasive, curatively intended treatment for various tumors, including hepatic, renal, and pulmonary. Emerging techniques like high-intensity focused ultrasound (HIFU) and irreversible electroporation (IRE) further expand the interventional toolkit, allowing for non-invasive and precise tumor ablation in certified cases, such as uterine myoma and prostate cancer [13].

The surgical dimension in oncology

The field of surgical oncology has undergone a substantial transformation, departing from extensive radical surgeries in the 20th century, which lacked solid scientific grounding. Initially, these practices were influenced by concepts like Halsted's, which primarily regarded cancer as a localized issue. However, a shift occurred in the mid-20th century as research, clinical trials, and scientific discoveries contradicted these notions. Cancer was increasingly recognized as a complex, systemic condition involving intricate interactions between the tumor and the host, with the bloodstream playing a crucial role in metastasis. As a result, there was a growing emphasis on less aggressive surgeries, accompanied by post-operative systemic therapies, ultimately enhancing patient outcomes [14].

Surgery excels in the early cancer stages, offering cost-effective curative treatment in one visit, even in resource-limited settings. Although its efficacy declines in advanced cancer, it can improve both life quality and duration, especially for conditions like malignant bowel obstruction and fungating breast cancers [5]. Many early solid tumors hold the potential for surgical cure, yet a significant portion of advanced cancer patients rely on surgery for symptom relief [15]. Minimally invasive surgeries have emerged as a significant advancement in the field of oncology, offering benefits such as equivalent and potentially superior advantages in select surgical outcomes without compromising oncologic outcomes. However, further research is needed to assess their impact on functional outcomes and quality of life [16].

Surgical intervention in metastatic cancers presents unique challenges. While surgery can benefit patients with metastatic colorectal, renal, and ovarian cancers, it is often avoided in cases of metastatic lung, pancreatic, and prostate cancer. The surgical removal of the primary tumor has the potential to boost the body's anti-cancer immune response, limit the spread of cancer cells, and make systemic treatments more effective, offering promise for better patient outcomes. Despite clinical evidence supporting the role of surgery in specific metastatic scenarios, real-world practice sometimes lags, with discrepancies between evidence and utilization. Metastatic cancer accounts for a substantial global burden of morbidity and mortality, necessitating a thorough exploration of the potential benefits of primary site surgery in such cases [17].

The importance of post-surgical care and rehabilitation in oncology cannot be overstated. Cancer rehabilitation is gaining recognition as a crucial component of cancer care. Effective exercise prescription and rehabilitation can significantly reduce hospital stay length, and post-operative complications, and enhance the recovery and quality of life for cancer patients following surgery [18].

Integrating medicine and surgery in oncology

Integrating medicine and surgery in oncology has become a crucial approach in the modern treatment of cancer. Neoadjuvant and adjuvant therapies have emerged as valuable tools in bridging the gap between medicine and surgery, leading to more effective patient outcomes. Neoadjuvant therapy, initially defined as systemic treatment before local intervention, has evolved to encompass preoperative chemotherapy and radiation. This approach offers various advantages, such as enhanced local and distant control, direct assessment of tumor response, and the potential for organ-sparing treatments. While neoadjuvant therapy has been established as a standard of care for certain cancer types, its utility in others continues to be explored through clinical trials, ensuring that it is compared closely to conventional treatments in terms of recurrence, survival, and organ preservation, as well as quality of life and cost-effectiveness [19].

Adjuvant therapies play a pivotal role in refining cancer treatment strategies. Combination chemotherapy, widely embraced, is instrumental in enhancing therapeutic efficacy and reducing adverse effects across diverse cancer types. Concurrently, researchers are delving into the potential of botanical extracts and compounds, such as Solanum nigrum, Claviceps purpurea, curcumin, resveratrol, and matairesinol, when integrated with anti-tumor drugs to combat therapy resistance and offer protective benefits [20]. Moreover, adjuvant psychological therapy emerges as a promising avenue for mitigating multifaceted psychological distress encountered by cancer patients [21].

Multidisciplinary tumor boards (MTBs) have emerged as a collaborative decision-making platform where specialists and healthcare professionals come together to discuss cancer cases. These meetings facilitate the exchange of knowledge, expertise, and insights, leading to more comprehensive and effective treatment strategies. Studies have consistently shown that MTBs correlate with improved treatment outcomes and patient care. This multidisciplinary approach is an essential component of integrated oncology, ensuring that patients receive the most well-rounded care and personalized treatment plans [22].

Furthermore, a holistic approach to cancer management is increasingly recognized as a complementary element to conventional oncology. Integrative medicine combines evidence-based complementary therapies, lifestyle interventions, and conventional treatments to optimize health, reduce modifiable risk factors, and address the physical and emotional aspects of cancer. Such an approach may include herbal medicine, stress reduction practices, supplements, and physical therapies tailored to the individual patient's needs. By addressing modifiable factors like stress, poor nutrition, physical inactivity, and vitamin deficiencies, oncologists can contribute to better overall patient health and improved treatment outcomes [23].

Innovations and technological advancements

Innovations and technological advancements have played a pivotal role in reshaping the landscape of modern healthcare, particularly in the realm of oncology. One such groundbreaking development is the integration of robotic surgeries in the field of oncology. Robotic surgery has evolved into a minimally invasive surgical technique that offers numerous advantages over traditional open surgeries and laparoscopy. Its three-dimensional vision, magnification capabilities, and stable operating field empower surgeons to perform complex procedures with greater precision and minimal damage to critical structures. This translates to benefits for patients, including reduced scarring, minimized blood loss, quicker recovery times, fewer wound-related complications, and shorter hospital stays [24].

In cancer care, biomedical imaging techniques are indispensable, offering insights into tissue morphology, structure, metabolism, and functionality [25]. These techniques, when combined with other diagnostic methods, aid clinical decision-making. Hybrid imaging methods enhance cancer staging and treatment planning accuracy. Image-guided minimally invasive therapies have the potential to improve treatment outcomes and reduce collateral effects [25]. Computed tomography (CT) and magnetic resonance imaging (MRI) are advanced imaging technologies that have revolutionized surgery and disease management, enabling precise visualization of bones and soft tissue. They enhance surgical approaches for various medical conditions, including middle ear diseases, orthopedic procedures, reconstructive surgeries, and cancer treatments [26].

Personalized medicine, or precision medicine, is a game-changing advancement in oncology. By considering a patient's genetic makeup, tumor environment, lifestyle, and medical history, this approach tailors treatments precisely. It optimizes therapy, reduces side effects, preserves organ function, and elevates overall patient quality of life. It signifies a shift towards a more patient-centric and effective cancer care model [27].

Translational research has been instrumental in driving these innovations. Shifting from a one-size-fits-all approach to a molecular analysis-driven, personalized strategy has significantly improved the detection of predictive and prognostic molecular alterations in cancer. Technologies like next-generation sequencing and RNA sequencing have enabled the identification of gene mutations, amplifications, and fusions, profoundly impacting disease management in both localized and metastatic settings [28].

Challenges and controversies

Cancer, a diagnosis that brings forth a whirlwind of emotions, demands patients to navigate a labyrinth of challenges and controversies as they embark on their treatment journey. The paramount dilemma lies in balancing aggressive treatments with the quality of life. Patients grapple with the daunting prospect of potentially sacrificing their well-being for extended survival. The pursuit of increased overall and disease-free survival, the traditional focus of cancer treatment, now shares the spotlight with the recognition of the significance of Quality of Life (QoL) as a vital endpoint [29].

The ethical dimension of experimental cancer treatments, particularly Phase I clinical trials, presents unique challenges [30]. These trials, which evaluate pioneering treatment approaches, have evolved in response to advances in cancer biology, research methods, and ethical standards. Phase I trials entail elevated research requirements and uncertainties compared to other cancer studies, making their ethical considerations distinct. Patients often turn to Phase I trials when standard therapies are exhausted, and the outcomes of these trials significantly influence drug development and regulations, imposing ethical challenges in study design, informed consent, data disclosure, and therapy reductions while maintaining efficacy [31]. Collaboration and timely, high-quality data submission are vital, highlighting the ethical complexities inherent in oncology research and treatment.

Disparities in access to advanced oncological care present a grave issue. While cancer was once predominantly a disease of affluent populations, it has now become a leading cause of morbidity and mortality in low- and middle-income countries. Within this evolving landscape, inequalities have burgeoned, affecting socially disadvantaged population sub-groups. Factors like poor awareness, low socio-economic status, weak healthcare systems, and the overwhelming financial burden of cancer care contribute to these disparities, pushing disadvantaged patients and their families into a cycle of poverty [32].

Patient-centered care in oncology

Patient-centered care in oncology is an essential approach that focuses on the overall well-being and satisfaction of cancer patients and their families. This care model recognizes the importance of palliative care alongside aggressive treatments, aiming to improve the patient's symptoms, quality of life, mood, and even survival. Palliative care plays a central role in oncology, with clinical guidelines now recommending its integration early in the disease course, concurrent with active cancer treatment. By providing dedicated palliative care services, patients with advanced cancer can benefit from enhanced quality of life, less aggressive end-of-life care, and increased patient and caregiver satisfaction. This approach aligns with the critical need for healthcare services that can optimize patient outcomes without overburdening the healthcare system [33].

Psychological and emotional support is another vital component of patient-centered care in oncology. Cancer patients commonly experience persistent pain and psychological distress, and efficacious and timely psychological interventions are necessary to address these challenges. However, it is crucial to continue research to determine which interventions work best for specific cancer groups and outcomes. Methodological limitations must be addressed, and fully powered, head-to-head comparison trials are needed to enhance our understanding of the most effective psychological support strategies [34,35].

Informed decision-making is at the core of patient-centered care in oncology. Studies have consistently shown that patients who are well-informed about their condition, prognosis, and treatment options are more likely to adhere to treatments. Patients with cancer want access to evidence-based information about the likelihood of cure, disease spread, and treatment options to actively participate in decisions about their care [36]. Interest in informed decision-making has grown in recent years, aligning with recommendations to improve healthcare quality. Patients should have a comprehensive understanding of their condition, treatment benefits and risks, limitations, alternatives, and uncertainties, allowing them to make decisions that align with their preferences [37].

Future directions and predictions

The landscape of oncological care is poised for transformative changes in the coming decade, with a promising array of emerging therapies and technologies leading the way. Gene therapy, a groundbreaking approach involving the replacement of defective genes with healthy ones, is at the forefront of these advancements. Unlike traditional chemotherapy, gene therapy offers selective targeting, minimizing harm to healthy cells. Innovations in gene expression targeting have opened doors for tissue- and organ-specific treatments, positioning gene therapy as a potential first-line approach in the fight against cancer [38]. Stem cell therapy and nanoparticles hold promise for tissue regeneration and advanced diagnostics. Targeted therapy, ablation procedures, and natural antioxidants show potential in cancer treatment and prevention, while various emerging technologies are in clinical trial phases, with some already gaining approval [39].

Remote patient monitoring, another facet of the evolving oncological landscape, is set to revolutionize the way cancer patients are cared for. Outpatient treatment, while effective, has often left patients unmonitored for extended periods, particularly during the coronavirus disease 2019 (COVID-19) pandemic. Remote patient-reported outcome monitoring systems have emerged as a solution, showing benefits in terms of both economic and survival outcomes. These systems provide real-time data on patient well-being, enabling timely intervention and personalized care plans [40]. In parallel, the future of oncology care is also characterized by the rapid growth of tele-oncology, enabled by medical telecommunications. This technology not only enhances the accessibility of clinical cancer care but also serves as a powerful tool for education and training in the field of oncology. Tailoring the implementation of tele-oncology to suit the unique needs and resources of the developing world will be critical, and a programmatic, consistent, and long-term approach will be key to its success [41].

Looking ahead to the next decade, integrated oncological care is expected to be a driving force in improving patient outcomes. The integration of palliative care early in the disease course for patients with poor prognosis cancer has already demonstrated significant enhancements in various aspects, including quality of life, mood, patient satisfaction, prognostic understanding, health service use, and possibly survival. These pioneering studies challenge traditional oncology care models and raise essential questions about the mechanisms by which early palliative care improves patient outcomes, emphasizing the need for widespread dissemination of such models [42].

## Conclusions

Cancer treatment, historically marked by radicalism and limitations, has evolved into a patient-centered, integrated approach fueled by medical and surgical innovations. Medical oncology, with chemotherapy, targeted therapies, immunotherapy, and precision medicine, tailors treatments to individuals. Surgical oncology shifts from radical to minimally invasive techniques, emphasizing organ preservation and improved outcomes. Synergy in neoadjuvant, adjuvant therapies, and tumor boards ensures comprehensive care.

Innovations in robotics, imaging, and personalized medicine revolutionize cancer care. Challenges include ethical dilemmas in experimental treatments and global disparities. Patient-centered care, with palliative support and informed decisions, becomes essential. The future promises gene therapy, tele-oncology, remote monitoring, and early palliative care integration, highlighting the dynamic and transformative nature of oncological care and the unwavering commitment of the medical and surgical communities in the fight against cancer.
